# Macrostructure of *Malus* Leaves and Its Taxonomic Significance

**DOI:** 10.3390/plants14131918

**Published:** 2025-06-22

**Authors:** Yuerong Fan, Huimin Li, Jingze Ma, Ting Zhou, Junjun Fan, Wangxiang Zhang

**Affiliations:** 1 College of Forestry, Nanjing Forestry University, Nanjing 210037, China; fyr@njfu.edu.cn (Y.F.);; 2 Jiangsu Key Laboratory for the Research and Utilization of Plant Resources, Institute of Botany, Jiangsu Province and Chinese Academy of Sciences (Nanjing Botanical Garden Mem. Sun Yat-Sen), Nanjing 210014, China; 3 Jinling Institute of Technology, College of Horticulture, Nanjing 210038, China; joycefan2019@jit.edu.cn; 4 Co-Innovation Center for Sustainable Forestry in Southern China, Nanjing Forestry University, Nanjing 210037, China

**Keywords:** *Malus*, leaf architecture, numerical classification, ancestor-inclined distribution

## Abstract

Leaves are the most ubiquitous plant organs, whose macrostructures exhibit close correlations with environmental factors while simultaneously reflecting inherent genetic and evolutionary patterns. These characteristics render them highly significant for plant taxonomy, ecology, and related disciplines. Therefore, this study presents the first comprehensive evaluation of *Malus* leaf macrostructures for infraspecific classification. By establishing a trait-screening system, we conducted a numerical taxonomic analysis of leaf phenotypic variation across 73 *Malus* germplasm (34 species and 39 cultivars). Through ancestor-inclined distribution characteristic analysis, we investigated phylogenetic relationships at both the genus level and infraspecific ranks within *Malus*. A total of 21 leaf phenotypic traits were selected from 50 candidate traits based on the following criteria: high diversity, abundance, and evenness (D ≥ 0.50, H ≥ 0.80, and E ≥ 0.60); significant intraspecific uniformity and interspecific distinctness ($\bar{CV}$ ≤ 10% and CV ≥ 15%). Notably, the selected traits with low intraspecific variability ($\bar{CV}$ ≤ 10%) exhibit environmental robustness, likely reflecting low phenotypic plasticity of these specific traits under varying conditions. This stability enhances their taxonomic utility. It was found that the highest ancestor-inclined distribution probability reached 90% for 10 traceable cultivars, demonstrating reliable breeding lines. Based on morphological evidence, there was a highly significant correlation between the evolutionary orders of (Sect. *Docyniopsis* → Sect. *Sorbomalus* → Sect. *Malus*) and group/sub-groups (B_1_ → B_2_ → A). This study demonstrates that phenotypic variation in leaf macrostructures can effectively explore the affinities among *Malus* germplasm, exhibiting taxonomic significance at the infraspecific level, thereby providing references for variety selection. However, hybrid offspring may exhibit mixed parental characteristics, leading to blurred species boundaries. And convergent evolution may create false homologies, potentially misleading morphology-based taxonomic inferences. The inferred taxonomic relationships present certain limitations that warrant further investigation.

## 1. Introduction

Flowering crabapple (*Malus* spp.) refers to shrubs or small trees within the genus *Malus* of the Rosaceae family, characterized by fruit diameters of less than 5 cm [1]. China stands as the world’s largest gene center and genetic diversity hub for *Malus* species [2]. Globally, there are approximately 38 *Malus* species, with around 30 species found in China, 16 of which are endemic to the country [3]. According to statistics, there are approximately 200–700 cultivated crabapple varieties, encompassing both ornamental types and wild forms, which exhibit rich variations in tree architecture, flowers, leaves, and fruits [4]. Since the establishment of the genus Malus by Miller in 1754, the diversity and overlapping nature of morphological characteristics have led to severe issues of “synonymy” and “homonymy”, and the presence of numerous intermediate traits has made differentiation challenging, rendering *Malus* a notoriously “difficult genus” in plant taxonomy [5]. Regarding the taxonomy of the genus *Malus*, few researchers have explored the macrostructure of *Malus* leaves as a potential solution. Li et al. [6] revised the classification of wild *Malus* species based on herbarium specimens and numerical analysis. Liu et al. [7] supported the monophyly of *Malus* through nuclear phylogenetic analysis, while Forte et al. [8] inferred that section *Sorbomalus* comprises polyphyletic species using molecular DNA data. Previous studies have also employed molecular markers [9,10], isozymes [11], and palynology [12]. However, genetic distances estimated from different gene sequences often show inconsistencies [13]. And these investigations were limited by small sample sizes, focusing solely on species or varieties, and lacked the application of statistical methods to explore phylogenetic relationships. Ancestor-inclined distribution refers to the clustering of offspring and their parental lines through cluster analysis, thereby providing insights into their genetic relationships [14]. Building upon this methodological framework, Fan et al. [14] and Zhou et al. [15] conducted groundbreaking classification studies on *Malus* germplasm by analyzing floral organ and pollen phenotypic traits, respectively. However, no prior research has systematically applied this approach to the analysis of *Malus* leaf macrostructures.

Leaf macrostructures refer to the phenotypic characteristics of plant leaves that can be observed directly or under a dissecting microscope, such as leaf shape, size, pubescence, and venation patterns. These traits embody the plant’s genetic identity and phylogenetic evolutionary history, exhibiting distinct specificity at both genus and species levels [16]. Therefore, compared to molecular approaches, leaf macrostructures directly manifest outcomes of natural selection and ecological adaptation. Leaf venation is a regular network structure formed by interconnected vascular bundles and xylem components in plant leaves [17]. Research has shown that while leaf shape and size may vary among different individuals of the same species or across different parts of the same plant, the characteristics of higher-order venation patterns, vein branching forms, and areolation remain stable [18,19]. This stability suggests these traits are under strong genetic and phylogenetic constraints, making them valuable references for inferring evolutionary relationships and delineating both intergeneric and interspecific classifications. Currently, leaf macrostructures are widely utilized as significant taxonomic criteria in the classification studies of various plant groups, including the Sapindaceae [20], *Prunus* [21], *Sorbus* [22], *Berberis* [23], and *Cymbidium* [24]. However, current research on leaf macrostructures, particularly venation patterns, predominantly remains at the level of trait description [25]. Trait selection is primarily conducted through principal component analysis for dimensionality reduction, supplemented by correlation analysis and one-way ANOVA, lacking a scientific theoretical framework and systematic techniques for trait screening [26,27]. Furthermore, studies are often narrowly focused on the species or variety level, with classification results limited to germplasm identification and cluster group descriptions, failing to delve into the deeper phylogenetic and evolutionary relationships among germplasms [28].

This study investigates *Malus* germplasm through quantitative taxonomic and phylogenetic analyses of leaf macrostructures, aiming to (1) establish a theoretical and technical framework for screening phenotypic traits of *Malus* leaves; (2) evaluate the taxonomic significance of leaf macrostructure characteristics; (3) validate the grouping efficacy and infer evolutionary trends using the ancestor-inclined distribution analysis, thereby providing theoretical foundations for cultivar innovation and conservation of unique genotypes.

## 2. Results

### 2.1. Diversity and Variability of Leaf Phenotypic Traits in Malus Germplasm

**Figure 1:**
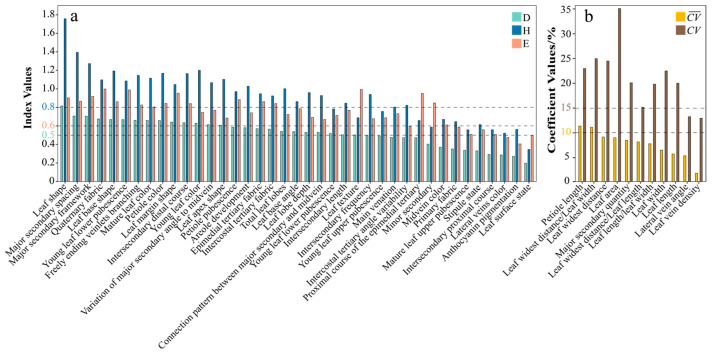
Diversity and variability analysis of leaf phenotypic traits in *Malus* germplasm. (**a**): the bar plot of Simpson’s Diversity Index (D), the Shannon–Wiener Information Index (H), and the Evenness Index (E) for qualitative traits. If the values of D are ≥0.50, H are ≥0.80, and E are ≥0.60, the trait is considered to meet the required criteria; (**b**) the bar plot of the mean coefficient of variation ($\bar{CV}$) and the coefficient of variation (CV) for quantitative traits. If $\bar{CV\;}$ is ≤10% and CV is ≥15%, the trait is considered to meet the required criteria.

The Simpson’s Diversity Index (D), Shannon–Wiener Information Index (H), and Evenness Index (E) for the qualitative traits of Malus leaves ranged from 0.20 to 0.82, 0.35 to 1.76, and 0.41 to 1.00, respectively (Figure 1a), indicating variations in diversity, richness, and evenness across different traits. These variations hold significant taxonomic implications: Traits with high D, H, and E indices exhibit balanced polymorphism and are more likely to reflect genetic divergence, serving as reliable markers for delineating subpopulation classifications [29]. Conversely, low-diversity traits may represent evolutionarily conserved characteristics shared across *Malus* germplasm, suggesting environmental insensitivity. Evenness (approaching 1.00) serves as a key indicator of taxonomic utility, with higher values enhancing resolution in phylogenetic grouping analyses.

For qualitative traits, 12 traits—leaf surface state, young leaf upper pubescence, mature leaf upper pubescence, anthocyanin pigmentation, stipule state, midvein color, main venation, lateral vein color, primary fabric, intersecondary proximal course, intercostal tertiary angle variability, and proximal course of the epimedial tertiary—exhibited D values below 0.50. Additionally, leaf texture and young leaf lower pubescence had H values below 0.8 (Figure 1a). Consequently, these low-diversity traits were excluded from the analysis. The remaining 24 qualitative traits demonstrated high diversity, rich variation information, and high evenness (D ≥ 0.50, H ≥ 0.80, and E ≥ 0.60) [30] and were retained. For quantitative traits, petiole length and leaf widest distance/leaf width showed mean coefficient of variation ($\bar{CV}$) values greater than 10%, while lateral vein angle and leaf vein density exhibited coefficient of variation (CV) values exceeding 15% (Figure 1b). Therefore, these traits have been excluded from the analysis. The remaining seven quantitative traits displayed significant intraspecific uniformity and interspecific distinctness ($\bar{CV}$ ≤ 10% and CV ≥ 15%) and were retained.

### 2.2. Principal Component Analysis of Leaf Phenotypic Traits in Malus Germplasm

Principal component analysis (PCA) was conducted on 31 selected leaf phenotypic traits to identify key classification characteristics through dimensionality reduction. Using an eigenvalue threshold of λ ≥ 0.90 [15], the first 12 principal components were extracted, accounting for 83.54% of the cumulative variance. Eigenvector absolute values > 0.60 were considered significant [6]. The eigenvector values of each trait in different principal components are presented in Table 1.

The first principal component (PC1) accounts for 15.53% of the total variance and primarily captures variation in leaf division and shape. PC2 explains 13.41% of the variance, predominantly reflecting leaf size, while PC3 (9.42%) primarily captures basal morphology. Collectively, the first three PCs demonstrate that leaf shape and size hold significant taxonomic value for *Malus* classification, encompassing substantial discriminatory information. In contrast, PCs 4–7 reveal a gradual shift in data structure from color and pubescence traits toward secondary and tertiary venation characteristics, indicating that vein architecture carries finer-scale taxonomic significance. PCs 8–10 characterize features of intersecondary veins, major secondary veins, and areolar morphology in *Malus* leaves, while PCs 11–12 primarily capture leaf apex and margin configurations. Consequently, low-contribution traits—including leaf length/leaf width, leaf shape, intersecondary length, intersecondary distal course, and major secondary framework—were excluded. The remaining 26 traits effectively capture the majority of information regarding the macroscopic structure of *Malus* leaves.

### 2.3. Pearson Correlation Analysis of Leaf Phenotypic Traits in Malus Germplasm

**Figure 2:**
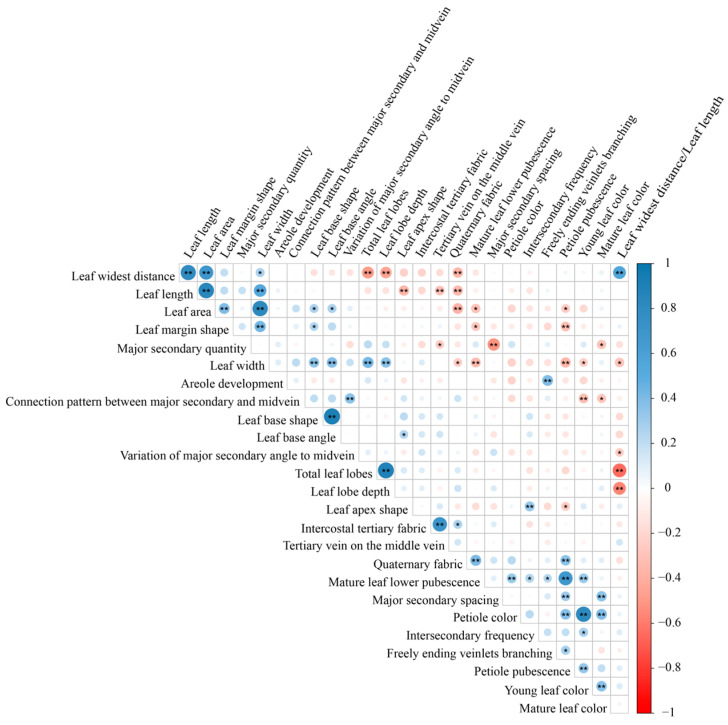
Pearson correlation analysis of leaf phenotypic traits in *Malus*. Note: ** indicates significant correlation at the 0.01 level (two-sided), and * indicates significant correlation at the 0.05 level (two-sided). Blue dots indicate positive correlations, red dots indicate negative correlations, and the size of the dots represents the magnitude of the correlation coefficient.

Following principal component analysis, 26 leaf phenotypic traits (21 qualitative and 5 quantitative) were selected for Pearson correlation analysis (Figure 2). Using a threshold of r > 0.80 [15], the results indicated that most leaf phenotypic traits were independent of each other, while a few exhibited highly significant (*p* < 0.01) correlations, such as leaf length and leaf widest distance (r = 0.81), leaf area and leaf length (r = 0.84), leaf width and leaf area (r = 0.83), leaf base angle and leaf base shape (r = 0.91), leaf lobe depth and total leaf lobes (r = 0.89), which reflect coordinated allometric growth during leaf development, as well as young leaf color and petiole color (r = 0.81), which may suggest shared biochemical pathways activated in young tissues (e.g., anthocyanin biosynthesis). Therefore, based on observational convenience, the traits of leaf widest distance, leaf area, leaf base shape, leaf lobe depth, and young leaf color were retained for quantitative classification.

### 2.4. Cluster Analysis of Leaf Phenotypic Traits in Malus Germplasm

**Figure 3:**
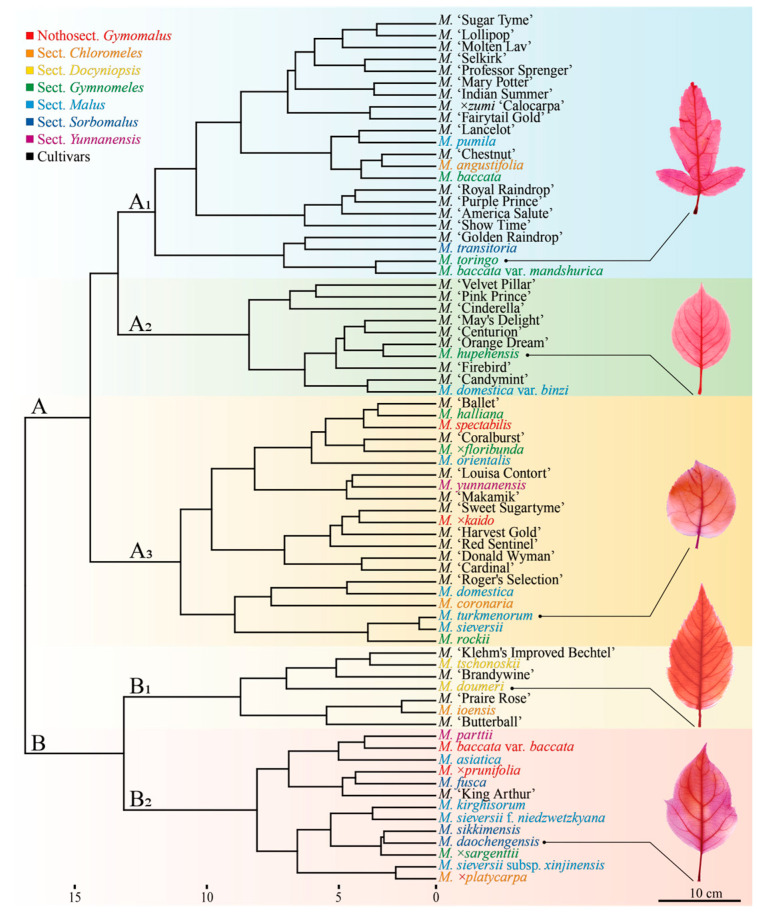
Clustering dendrogram of *Malus* germplasm. The scientific names of species are color-coded, with the same color indicating membership in the same group [5]. The color scheme is as follows: red font means species in Nothosect. *Gymomalus*; orange font means species in Sect. *Chloromeles*; yellow font means species in Sect. *Docyniopsis*; green font means species in Sect. *Gymnomeles*; cyan font means species in Sect. *Malus*; blue font means species in Sect. *Sorbomalus*; purple font means species in Sect. *Yunnanensis*; and black font means cultivars.

Following Pearson correlation analysis, 21 leaf phenotypic traits (18 qualitative and 3 quantitative) were selected for subsequent analysis. Using Euclidean distance and Ward’s D2 linkage, hierarchical clustering was performed on 73 germplasm accessions (Figure 3). At a genetic distance of 16.41, the 73 *Malus* germplasms were divided into two major groups (A and B). Group A comprised 53 germplasms, characterized primarily by green young leaves and densely pubescent petioles; Group B consisted of 20 germplasms, distinguished mainly by brown-red to purple-red young leaves and sparsely pubescent or glabrous petioles. Further subdivision at a genetic distance of 12.02 resulted in five distinct subgroups (A_1_, A_2_, A_3_, B_1_, and B_2_). The leaf quantitative traits exhibit significant differentiation among these subgroups (Table 2).

Subgroup A_1_: This cluster comprises 22 germplasm accessions (6 species and 16 cultivars). Key characteristics include major secondary spacing generally gradually or abruptly increasing proximally; intersecondary frequency typically being less than one per intercostal area; intercostal tertiary fabric being predominantly opposite percurrent; freely ending veinlets mostly having one branch or dendritic; a higher number of major secondary veins; a relatively short leaf widest distance; and the smallest leaf area.

Subgroup A_2_: This cluster consists of 10 germplasm accessions (2 species and 8 cultivars). Key characteristics include major secondary spacing generally gradually or abruptly increasing proximally; intersecondary frequency is typically one or more than one per intercostal area; intercostal tertiary fabric is opposite percurrent; freely ending veinlets are mostly with one branch; a higher number of major secondary veins; a longer leaf widest distance; and a relatively small leaf area.

Subgroup A_3_: This cluster comprises 21 germplasm accessions (11 species and 10 cultivars). Key characteristics include major secondary spacing that generally gradually increases proximally or is irregular; an intersecondary frequency that is typically less than one per intercostal area; an intercostal tertiary fabric that is predominantly mixed percurrent; freely ending veinlets that mostly have one branch or are unbranched; fewer major secondary veins; the shortest leaf widest distance; and a relatively large leaf area.

Subgroup B_1_: This cluster includes 7 germplasm accessions (3 species and 4 cultivars). Key characteristics include major secondary spacing that is entirely regular; an intersecondary frequency that is typically less than one per intercostal area; an intercostal tertiary fabric that is predominantly opposite percurrent; freely ending veinlets that mostly have one branch or are unbranched; the highest number of major secondary veins; a longer leaf widest distance; and a relatively large leaf area.

Subgroup B_2_: This cluster consists of 13 germplasm accessions (12 species and 1 cultivar). Key characteristics include major secondary spacing that generally gradually increases proximally; an intersecondary frequency that is typically less than one per intercostal area; an intercostal tertiary fabric that is predominantly opposite or alternate percurrent; freely ending veinlets that mostly have one branch; the fewest major secondary veins; the longest leaf widest distance; and the largest leaf area.

### 2.5. Analysis of Ancestor-Inclined Distribution Characteristic in Malus Germplasm

The classification system of the genus *Malus* primarily falls into two categories: one, represented by Koehne [31], groups species based on the persistence of fruit calyx; the other, more influential system, proposed by Rehder [32], classifies species according to leaf lobing. This system has been further refined by Chinese scholars such as Yu Dejun [33], Li Yunong [34], and Qian Guanze [5]. Based on Qian Guanze’s [5] revised system, the 34 *Malus* species in this study belong to seven sections (Figure 3): Sect. *Docyniopsis* (2 species), Sect. *Yunnanensis* (2 species), Sect. *Sorbomalus* (4 species), Sect. *Chloromeles* (4 species), Sect. *Malus* (10 species), Sect. *Gymnomeles* (8 species), and Nothosect. *Gymomalus* (4 species).

The distribution of these seven sections across the two major clusters (A and B) was relatively balanced, accounting for 55.9% and 44.1%, respectively. Within the five subgroups, the species were predominantly distributed in A_3_ (32.4%) and B_2_ (35.3%). Specifically, Sect. *Docyniopsis* was found exclusively in subgroup B_1_, while Sect. *Yunnanensis* was evenly distributed between A_3_ (50%) and B_2_ (50%). Sect. *Sorbomalus* showed an uneven distribution, with 25% in A_1_ and 75% in B_2_. Sect. *Chloromeles* was evenly distributed across four subgroups (A_1_, A_3_, B_1_, and B_2_, each 25%). Sect. *Malus* exhibited an uneven distribution, with 10% in A_1_, 10% in A_3_, 40% in B_1_, and 40% in B_2_. Sect. *Gymnomeles* was unevenly distributed across A_1_ (37.5%), A_2_ (12.5%), A_3_ (37.5%), and B_2_ (12.5%). Finally, Nothosect. *Gymomalus* was evenly distributed between A_3_ (50%) and B_2_ (50%).

The distribution proportions of the aforementioned *Malus* species across the five subgroups are ranked as follows: B_2_ (92.3%) > A_3_ (52.4%) > B_1_ (42.9%) > A_1_ (27.3%) > A_2_ (20.0%). Given the relatively low proportions of *Malus* species in subgroups A1 and A2, these were merged with A3 to form a consolidated group A. We hypothesize that the evolutionary sequence of the three *Malus* germplasm groups (A, B_1_, B_2_) may be B_1_ → B_2_ → A. Aligning this with the evolutionary sequence of the three sections in the classical classification system (Sect. *Docyniopsis* → Sect. *Sorbomalus* → Sect. *Malus*) [35], we assigned values to these groups: B_1_ (1) → B_2_ (2) → A (3); Sect. *Docyniopsis* (1) → Sect. *Sorbomalus* (2) → Sect. *Malus* (3). The results revealed a highly significant correlation between these two sets of evolutionary data (R^2^ = 0.713, *p* < 0.01).

According to the literature [36,37,38], among the 39 tested *Malus* cultivars, 10 could be traced back to their full or partial parental origins, involving 7 parental species (*Malus. baccata*, *M. baccata* var. *mandshurica*, *M.* ×*floribunda*, *M. halliana*, *M. ioensis*, *M. sieversii* f. *niedzwetzkyana*, and *M. toringo*). These 10 cultivars exhibited distinct ancestor-inclined distribution characteristics across the two major groups (A, B) and the five subgroups (A_1_, A_2_, A_3_, B_1_, and B_2_), with ancestor-inclined distribution probabilities reaching 90.0% and 80.0%, respectively (Table 3). The breeding frequency of the seven parental species, ranked from highest to lowest, was as follows: *M. toringo* (35.0%) > *M. halliana*, *M. ioensis*, *M. sieversii* f. *niedzwetzkyana* (15.0%) > *M. baccata* var. *mandshurica* (10.0%) > *M. baccata*, *M.* × *floribunda* (5.0%).

## 3. Discussion

### 3.1. Screening System for Macrostructural Classification Traits of Malus Leaves

Among plant organs, leaves exhibit the greatest number of traits [39]. During plant evolution, leaves display considerable plasticity and are highly susceptible to environmental factors such as light, temperature, and moisture [40]. Variations in leaf shape, epidermal characteristics, and anatomical structures can occur not only between species but also within species [41], highlighting the diversity and multidimensional nature of leaf macrostructures. However, an excessive number of variable dimensions can obscure the specificity of different subjects. Therefore, selecting representative traits is a critical aspect of numerical classification [42].

The study established a theoretical and technical framework for screening taxonomic leaf traits of *Malus* leaves: traits with low diversity and evenness were first eliminated through diversity and variability analysis → low-contribution traits were then removed via principal component analysis → strongly correlated traits were further excluded through correlation analysis. This process effectively identified 21 key leaf phenotypic traits. These traits collectively reflect the color, pubescence, shape, and size of *Malus* leaves, as well as the shape, number, structure, and development of leaf veins.

Consistent with this study, Chu et al. [43] conducted numerical classification on 28 *M. halliana* cultivars in Henan Province, finding that traits such as young leaf color and pubescence at flowering could serve as criteria for cultivar classification within the *M. halliana* group. Similarly, Feng et al. [28] compared the morphological characteristics of 25 *Osmanthus fragrans* cultivars (clones), identifying mature leaf color, leaf cross-section, and leaf width as primary classification criteria. This study further demonstrates that venation-related traits remain applicable for classification at the infraspecific level. This study provides supplementary morphological evidence for traditional taxonomy, advances quantitative systematics through leaf trait quantification, and establishes a theoretical and technical framework for screening phenotypic classification traits of *Malus* leaves. The developed system offers significant guidance for extracting macrostructural features of plant leaves.

### 3.2. Taxonomic Significance of Malus Leaf Macrostructures

Leaf venation, a two-dimensional branching network in plant leaves [44], plays a critical role in water transport efficiency, gas exchange rates, and overall plant performance [45]. Its structural organization reflects significant environmental adaptation strategies [46]. Strong constraints on environmental resources arise from selective pressures on leaf shape and function [17]. Studying the variation in leaf macrostructures can reveal interactions between genetics and the environment, reflecting the genetic patterns and extent of variation within populations.

In this study, cluster analysis based on the macrostructures of leaves from 73 *Malus* germplasms revealed that species within the same section were relatively concentrated in their distribution. The hypothesized evolutionary sequence of the three *Malus* germplasm groups (B_1_ → B_2_ → A) showed a highly significant correlation (R^2^ = 0.713, *p* < 0.01) with the evolutionary data of the three sections (Sect. *Docyniopsis* → Sect. *Sorbomalus* → Sect. *Malus*) in the classical classification system. Thus, the classification results of this study align well with the classical system represented by Rehder, providing insights into the evolutionary relationships among some sections. The study reveals that apparent inconsistencies between morphological and genetic data have emerged in recent taxonomic research [6]. At the species level, the clustering results demonstrate strong concordance with molecular phylogenetic studies:

Sect. *Gymnomeles*: *M. hupehensis* and *M. domestica* var. *binzi* cluster in A_2_, matching Zhou et al.’s [15] floral trait analyses. *M. baccata* and *M. toringo* form A_1_, corroborating Cho et al.’s [47] plastome characterization and comparative analyses evidence.

Sect. *Malus*: *M. orientalis*, *M. domestica*, and *M. sylvestris* group in A_3_, consistent with Liu et al. [7] and Höfer et al. [27]’s nuclear phylogenies.

Sect. *Docyniopsis*: *M. tschonoskii* and *M. doumeri* (primitive members) cluster in B_1_, aligning with Li et al.’s [6] and Forte et al.’s [8] molecular data.

Sect. *Sorbomalus*: *M. fusca*, *M. daochengensis*, and *M. parttii* form B_2_, supporting Forte et al.’s [8] hypothesis of their close affinity to Sorbomalus. Furthermore, only *M. transitoria* clustered within group A1, suggesting that the Sect. *Sorbomalus* likely comprises polyphyletic species. These validate that leaf macrostructures can be a reliable proxy for species delimitation in *Malus*, particularly when integrated with molecular data. However, certain discrepancies with molecular data persist—notably, the unexpectedly distant relationship between *M. sieversii* f. *niedzwetzkyana* and *M. domestica*—which warrant further investigation through multi-omics approaches.

At the cultivar level, among the 10 cultivars with fully or partially traceable parental origins, a clear ancestor-inclined distribution pattern was observed across the two major groups (A, B) and the five subgroups (A_1_, A_2_, A_3_, B_1_, B_2_), with ancestor-inclined distribution probabilities reaching 90.0% and 80.0%, respectively. This indicates that leaf macrostructures can be effectively applied to analyze genetic relationships among *Malus* germplasms. These findings align with the results of this study, suggesting that leaf macrostructures can serve as a reliable method for identification within genera or among species.

### 3.3. Evolutionary Trends in Malus Leaf Macrostructure and Breeding Implications

Phenotypic data analysis revealed that the most primitive group, B_1_, exhibited densely pubescent lower leaf surfaces, regular spacing of major secondary veins, and a high number of major secondary veins, with highly significant differences (*p* < 0.01) compared to the other four groups. Wang et al. [48] observed a trend from simple to complex in the surface ornamentation of pollen exine of Subgen. *Yulania*. Zhou et al. [49] discovered that crabapple floral organs exhibit a progressive decline in structural regularity from the species level to the cultivar level. Based on this, we hypothesize that the evolutionary trend of *Malus* leaf macrostructures is as follows: dense → sparse lower pubescence, high → low number of major secondary veins, and regular → irregular spacing of major secondary veins. Patterns of trait variation across resource and environmental gradients (light, water, nutrients, and temperature) probably reflect adaptation [50]. From an ecological functional perspective, the inferred evolutionary trend may reflect *Malus* taxa’s gradual adaptation to more stable, humid, and temperate environments, reducing selective pressures for defense and tolerance against extreme physical conditions (drought, intense light, high temperatures, strong winds). This adaptation manifests morphologically as decreased resource allocation to physical defense structures (pubescence) and costly hydraulic/support systems (number of major secondary veins). Notably, the selected traits exhibiting low intraspecific variability ($\bar{CV}$ ≤ 10%) demonstrate environmental insensitivity, likely reflecting limited phenotypic plasticity of these specific traits across varying conditions. However, this study has certain limitations: leaf macrostructure may vary with age or growth conditions, the lack of molecular validation, potential blending of parental traits in hybrid offspring obscuring species boundaries, and convergent evolution potentially creating false homologies that may mislead morphology-based taxonomy. Consequently, the inferred taxonomic relationships should be interpreted with caution.

For a long time, ornamental crabapples have been important woody flowering plants in landscape design. Research on crabapple flower color [51], flowering period [4], and floral fragrance [52] has been relatively well established. With the continuous advancement of breeding efforts, variegated foliage varieties of ornamental crabapples, such as ‘Duojiao’ [53] and ‘Datang Tingliang’ [54], have emerged in recent years, providing additional dimensions for exploring ornamental traits in crabapples. This study found that cultivars with young leaves ranging from brown-red to purple-red, strong anthocyanin pigmentation, and red to purple petioles, including *M*. ‘Centurion’, *M*. ‘May’s Delight‘, and *M*. ‘Pink Prince’, clustered into a distinct group (A_2_). Building upon previous studies of ancestor-inclined distribution in floral organs [15] and pollen [14], we hypothesize that certain macrostructural leaf traits in *Malus* may be heritable across generations, with offspring exhibiting significant ancestor-inclined distribution patterns in cluster analyses. Breeding Implications: Selecting cultivars with vibrant leaf coloration from cluster A_2_ as parental lines for hybridization could facilitate the development of new ornamental-leaf cultivars. This strategy provides a targeted breeding framework, leveraging heritable leaf traits to achieve desired aesthetic characteristics. In breeding practices, it is essential to account for dominant/recessive inheritance patterns, polygenic control mechanisms, and the selection of parental lines based on pigment metabolic pathways. The study reveals that chlorophyll determines the fundamental coloration of *Malus* leaves [55]. During leaf development, chlorophyll and carotenoid content progressively increase while anthocyanin levels decrease, with chlorophyll concentrations consistently surpassing other pigments [56]. This dynamic results in the gradual greening of mature leaves and produces unstable color transition phases. Therefore, future research should integrate classification traits with ornamental traits, such as the duration and extent of color transition, to provide a more robust theoretical foundation for the breeding of foliage-focused crabapple varieties.

## 4. Materials and Methods

### 4.1. Experimental Site and Materials

A total of 73 *Malus* germplasms (34 species and 39 varieties) (Table 4) were used in this study, all sourced from the national repository of *Malus* germplasm in Yangzhou (Jiangdu District, Yangzhou City, Jiangsu Province, China, 119°55′ E, 32°42′ N). The trees, aged between 7 and 10 years, had reached the flowering and maturity stage.

### 4.2. Trait Measurement, Description, and Encoding

The selection of leaf macrostructure traits was conducted with reference to the *Manual of Leaf Architecture* [57]. Additionally, traits were further selected and supplemented based on their distinctiveness and recognizability. A total of 50 phenotypic traits of *Malus* leaves were selected, including 39 qualitative traits and 11 quantitative traits. From 2021 to 2024 (May to July), these traits were observed and recorded repeatedly over four consecutive years for validation and correction. Standard specimens (healthy trees with uniform growth vigor) were selected following the principles of typicality, standardization, and consistency. For each germplasm, a minimum of five randomly selected trees were sampled. From the sun-exposed, current-year shoots of each tree, mature leaves were collected from the mid-canopy region and pooled to form a composite sample of 30 leaves representing that germplasm. The leaf venation specimens of *Malus* were prepared using the clearing method [16]. After preparation, three complete leaf venation specimens from each germplasm were selected for observation and recording of traits using a stereomicroscope (Leica DM 5000 B, Germany), followed by scanning and photography. Quantitative traits of the leaves were measured using ImageJ software (version 1.53). Qualitative traits were encoded using ordinal numerical coding, employing a consecutive series of non-negative integers (0, 1, 2, 3, …). Binary traits were typically assigned 0 for “no” and 1 for “yes” [28]. Quantitative traits were directly used in numerical form for subsequent analysis (Table 5).

### 4.3. Selection of Categorical Traits

(1) Qualitative Traits: The diversity, richness, and evenness of qualitative traits were assessed using Simpson’s Diversity Index (D), the Shannon–Wiener Information Index (H), and the Evenness Index (E). Traits were considered to meet the criteria of diversity, richness, and evenness if D ≥ 0.50, H ≥ 0.80, and E ≥ 0.60 [30].

(1) $$D=1-\sum_{i=1}^{k}\frac{n_{i}\left(n_{i}-1\right)}{n\left(n-1\right)}$$

(2) $$H=-\sum_{i=1}^{k}\frac{n_{i}}{n}\ln\frac{n_{i}}{n}$$

(3) $$E=\frac{H}{H_{k}}$$

where n represents the total number of germplasms, $n_{i}$ denotes the number of germplasms classified into the i-th level of each trait, and k indicates the total number of levels for each trait.

(2) Quantitative Traits: The intraspecific uniformity and interspecific distinctness of quantitative traits were assessed using the mean coefficient of variation ($\bar{CV}$) and coefficient of variation (CV) as described by Zhou et al. [15]. Traits were considered to meet the criteria of significant intraspecific uniformity and interspecific distinctness if $\bar{CV}$ ≤ 10% and CV ≥ 15% [15].

(4) $$\bar{CV}=\frac{1}{n}\sum_{i=1}^{n}\frac{S_{i}}{\bar{X_{i}}}\times 100\%$$

(5) $$CV=\frac{S^{\prime }}{\bar{X^{\prime }}}\times 100\%$$

where n is the number of germplasms, $S_{i}$ and $\bar{X_{i}}$ represent the standard deviation and mean of observed values for each trait within a germplasm, while $S^{\prime }$ and $\bar{X^{\prime }}$ denote the standard deviation and mean of trait values across all germplasms.

(3) Principal Component and Correlation Analysis: The original data were standardized to eliminate dimensional differences. Principal component analysis and correlation analysis were then conducted on the selected traits to further reduce dimensionality.

### 4.4. Cluster Analysis and Ancestor-Inclined Distribution Characteristic

**Figure 4:**
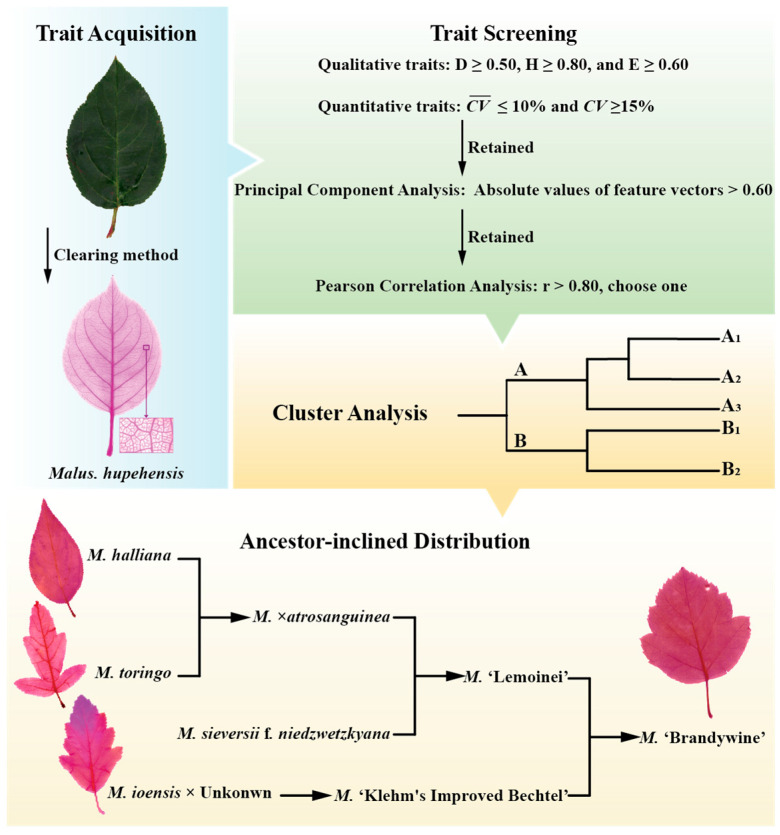
Outline of the study procedure.

The Euclidean distances of the screened leaf traits were calculated, followed by hierarchical clustering of *Malus* germplasms using the Ward.D2 linkage. The outline of the study procedure is shown in Figure 4. As shown in Figure 4, following the breeding pedigrees, when an offspring cultivar clusters within the same group/subgroup as one of its parental species, this indicates ancestor-inclined distribution. The ancestor-inclined distribution probability is calculated as the proportion of all pedigreed cultivars exhibiting this pattern.

### 4.5. Data Processing

Principal component analysis and correlation analysis were performed using SPSS Statistics 26 [58]. Data visualization was conducted using the R 4.3.1 program [59]. The bar charts were plotted using the “ggplot2” package [60], the correlation heatmap was generated with the “corrplot” package [61], and the cluster analysis plot was created using the “ggsci” package [62].

## 5. Conclusions

This study presents the first comprehensive evaluation of *Malus* leaf macrostructures for infraspecific classification. Many scholars have investigated the origins and phylogenetic relationships of *Malus* species using herbarium specimens, numerical analyses, nuclear phylogenetics, and DNA sequencing—yet largely overlooked the critical taxonomic value of leaf macrostructure. This study established a theoretical and technical system for screening leaf phenotypic classification traits in *Malus*, which can effectively reduce the dimensionality of numerous leaf phenotypic traits and identify traits that are high diversity, abundance, and evenness, as well as significant intraspecific uniformity and interspecific distinctness. These traits can serve as a reliable basis for the classification of *Malus* germplasm. In the cluster analysis, species within the same group exhibited relatively concentrated distributions, and traceable cultivars showed clear ancestor-inclined distribution characteristics. The hypothesized evolutionary sequence of the three groups (B_1_ → B_2_ → A) showed a highly significant correlation with the evolutionary data of the three sections (Sect. *Docyniopsis* → Sect. *Sorbomalus* → Sect. *Malus*) in the classical classification system. Therefore, the phenotypic variation in *Malus* leaf macrostructures can effectively elucidate phylogenetic relationships within the genus *Malus*, providing novel perspectives for infraspecific taxonomic studies. The diversity of young leaf colors offers valuable references for ornamental leaf-type cultivar selection. However, this study has certain limitations: the lack of molecular validation, potential blending of parental traits in hybrid offspring obscuring species boundaries, and convergent evolution potentially creating false homologies that may mislead morphology-based taxonomy. Consequently, the inferred taxonomic relationships should be interpreted with caution. Future studies should integrate molecular phylogenetics and controlled horticultural experiments to evaluate phenotypic plasticity and decipher complex hybridization histories.

## Figures and Tables

**Table 1:** Characteristic value, contribution rate, and cumulative contribution rate of each principal component.

Phenotypic Traits of Leaves	Principal Component											
	1	2	3	4	5	6	7	8	9	10	11	12
Total leaf lobes	**0.95**	−0.07	−0.05	−0.09	0.04	−0.07	0.02	−0.07	−0.02	0.04	0.09	0.01
Leaf lobe depth	**0.93**	−0.05	−0.08	0.07	0.01	0.04	−0.01	0.01	−0.07	0.03	0.10	0.03
Leaf widest distance/Leaf length	**−0.72**	0.09	−0.24	−0.05	0.06	0.15	0.08	0.09	−0.29	0.01	0.27	0.19
Leaf length/leaf width	−0.56	0.13	−0.36	0.28	0.17	−0.24	−0.22	0.02	0.00	0.01	−0.38	−0.29
Leaf shape	−0.49	−0.10	−0.35	0.02	0.12	0.00	0.02	0.01	−0.29	0.13	0.37	−0.07
Leaf length	−0.08	**0.93**	−0.03	0.02	0.01	−0.09	−0.17	−0.08	0.05	0.02	−0.22	−0.03
Leaf area	0.03	**0.92**	0.23	−0.19	−0.10	0.02	−0.01	−0.09	0.07	−0.01	0.03	0.13
Leaf widest distance	−0.48	**0.81**	−0.19	−0.02	0.04	0.02	−0.10	−0.04	−0.12	0.01	0.00	0.10
Leaf width	0.47	**0.69**	0.31	−0.24	−0.12	0.10	0.10	−0.06	0.08	0.04	0.10	0.22
Leaf base shape	0.02	0.05	**0.95**	0.01	−0.02	0.00	0.06	0.07	0.02	−0.06	0.05	0.10
Leaf base angle	0.06	0.09	**0.92**	0.01	0.04	−0.08	0.09	0.04	0.04	−0.05	0.11	0.07
Petiole pubescence	−0.12	0.00	−0.09	**0.81**	0.21	0.15	0.00	0.12	−0.09	−0.01	−0.12	−0.18
Mature leaf lower pubescence	−0.01	−0.12	0.10	**0.78**	0.18	−0.09	0.00	0.13	−0.12	0.08	−0.11	−0.18
Quaternary fabric	0.13	−0.40	0.03	**0.64**	0.08	0.14	0.15	−0.15	0.14	0.03	0.06	0.23
Young leaf color	−0.07	−0.04	−0.05	0.15	**0.90**	−0.08	−0.01	0.21	−0.02	−0.08	0.03	−0.05
Petiole color	0.00	−0.09	0.00	0.25	**0.86**	−0.06	−0.02	0.08	−0.02	−0.11	0.02	0.03
Mature leaf color	0.06	0.08	0.10	−0.02	**0.61**	0.58	−0.02	−0.19	−0.23	0.02	−0.07	−0.01
Major secondary spacing	0.07	0.07	−0.11	0.26	0.00	**0.82**	−0.04	0.03	0.12	−0.04	−0.03	−0.03
Major secondary quantity	0.17	0.12	−0.01	0.13	0.12	**−0.81**	−0.18	−0.07	−0.11	0.07	−0.03	0.06
Epimedial tertiary fabric	−0.10	−0.13	0.11	−0.05	−0.06	0.07	**0.91**	0.06	−0.02	−0.01	0.06	0.00
Intercostal tertiary fabric	0.13	−0.04	0.06	0.12	0.02	0.05	**0.89**	−0.08	0.12	−0.09	−0.02	0.04
Intersecondary frequency	−0.10	−0.07	0.16	0.12	0.17	0.03	−0.13	**0.78**	0.00	0.02	0.17	−0.15
Intersecondary length	0.49	−0.14	−0.11	−0.02	0.13	−0.03	0.28	0.59	−0.15	0.11	−0.12	0.21
Intersecondary distal course	−0.43	−0.13	0.08	0.11	0.05	0.08	0.03	0.58	−0.24	−0.07	0.10	0.34
Variation of major secondary angle to midvein	0.09	0.00	−0.02	−0.17	0.00	0.17	0.12	−0.04	**0.83**	0.02	−0.12	−0.03
Connection pattern between major secondary and midvein	−0.05	0.09	0.19	0.19	−0.29	−0.03	−0.01	−0.23	**0.62**	0.10	0.45	0.14
Areole development	0.09	0.01	−0.05	−0.09	−0.09	−0.08	−0.04	−0.13	−0.03	**0.88**	−0.01	0.08
Freely ending veinlets branching	−0.01	0.03	−0.12	0.33	−0.09	−0.03	−0.10	0.26	0.16	**0.67**	−0.05	−0.17
Major secondary framework	−0.26	−0.03	0.37	0.08	−0.24	0.27	0.11	0.16	−0.22	0.38	−0.20	−0.28
Leaf apex shape	0.06	−0.17	0.20	−0.28	0.07	−0.05	0.04	0.26	−0.04	−0.13	**0.78**	−0.13
Leaf margin shape	−0.01	0.25	0.19	−0.23	−0.04	−0.11	0.04	0.03	0.02	−0.03	−0.10	**0.79**
Eigen value	4.82	4.16	2.92	2.56	2.27	2.04	1.61	1.48	1.18	1.00	0.96	0.90
Variance contribution rate	15.53	13.41	9.42	8.27	7.33	6.57	5.21	4.78	3.80	3.23	3.08	2.91
Cumulative contribution rate	15.53	28.95	38.36	46.63	53.97	60.53	65.74	70.52	74.32	77.55	80.63	83.54

Note: Bold numbers indicate traits with larger absolute values of retained feature vectors.

**Table 2:** Leaf quantitative traits in *Malus* subgroups based on cluster analysis.

Quantitative Traits	Subgroups				
	A_1_	A_2_	A_3_	B_1_	B_2_
Major secondary quantity	7.89 ± 1.37 b	7.21 ± 1.16 bc	6.94 ± 0.98 c	9.90 ± 1.93 a	6.72 ± 0.78 c
Leaf widest distance (cm)	2.96 ± 0.68 b	3.24 ± 0.75 b	2.95 ± 0.66 b	3.31 ± 0.64 b	4.17 ± 0.56 a
Leaf area (cm^2^)	23.26 ± 6.78 b	24.98 ± 7.72 b	26.59 ± 9.23 b	35.06 ± 8.07 a	39.75 ± 8.01 a
Leaf widest distance/Leaf length	0.38 ± 0.07 b	0.43 ± 0.04 a	0.41 ± 0.06 ab	0.38 ± 0.03 ab	0.42 ± 0.05 a

Note: The different lowercase letters indicate significant differences (*p* < 0.05) in each quantitative trait among different subgroups.

**Table 3:** Parent traceability and identification of ancestor-inclined distribution characteristics of *Malus* cultivars.

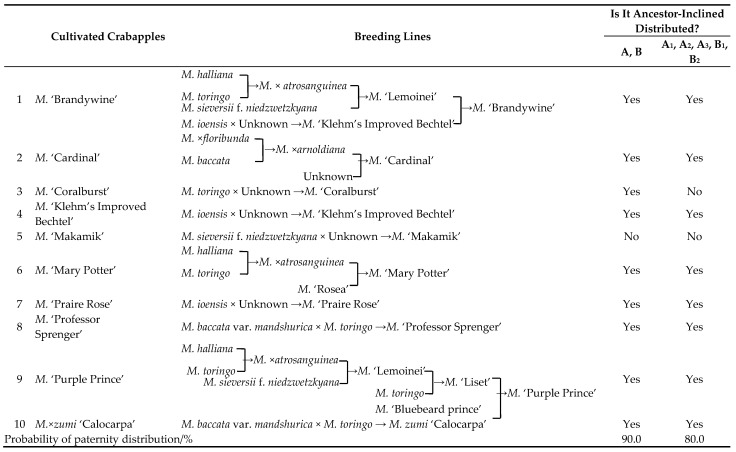

**Table 4:** The list of *Malus* germplasm.

Species	Species	Cultivars	Cultivars
*Malus angustifolia*	*M.* × *prunifolia*	*M.* ‘America Salute’	*M.* ‘Louisa Contort’
*M. asiatica*	*M. pumila*	*M.* ‘Ballet’	*M.* ‘Makamik’
*M. baccata*	*M. rockii*	*M.* ‘Brandywine’	*M*. ‘Mary Potter’
*M. baccata* var. *baccata*	*M.* × *sargenttii*	*M.* ‘Butterball’	*M.* ‘May’s Delight’
*M. baccata* var. *mandshurica*	*M. sieversii* f. *niedzwetzkyana*	*M*. ‘Candymint’	*M*. ‘Molten Lav’
*M. coronaria*	*M. sieversii* subsp. *xinjinensis*	*M.* ‘Cardinal’	*M.* ‘Orange Dream’
*M. daochengensis*	*M. sikkimensis*	*M.* ‘Centurion’	*M*. ‘Pink Prince’
*M. domestica*	*M. spectabilis*	*M.* ‘Chestnut’	*M.* ‘Praire Rose’
*M. domestica* var. *binzi*	*M. sylvestris*	*M.* ‘Cinderella’	*M*. ‘Professor Sprenger’
*M. doumeri*	*M. toringo*	*M.* ‘Coralburst’	*M.* ‘Purple Prince’
*M.* ×*floribunda*	*M. transitoria*	*M.* ‘Donald Wyman’	*M.* ‘Red Sentinel’
*M. fusca*	*M. tschonoskii*	*M.* ‘Fairytail Gold’	*M.* ‘Roger’s Selection’
*M. halliana*	*M. turkmenorum*	*M.* ‘Firebird’	*M.* ‘Royal Raindrop’
*M. hupehensis*	*M. yunnanensis*	*M.* ‘Golden Raindrop’	*M*. ‘Selkirk’
*M. ioensis*		*M.* ‘Harvest Gold’	*M.* ‘Show Time’
*M.* ×*kaido*		*M*. ‘Indian Summer’	*M*. ‘Sugar Tyme’
*M. kirghisorum*		*M.* ‘King Arthur’	*M.* ‘Sweet Sugar tyme’
*M. orientalis*		*M.* ‘Klehm’s Improved Bechtel’	*M*. ‘Velvet Pillar’
*M. parttii*		*M.* ‘Lancelot’	*M.*× *zumi* ‘Calocarpa’
*M.* ×*platycarpa*		*M.* ‘Lollipop’	

**Table 5:** Description and encoding of phenotypic traits in *Malus* leaves.

Leaf Phenotypic Traits	Description (Grades) and Encoding
Leaf texture	Papery: 0; Leathery: 1
Leaf surface state	Flat: 0; Wavy: 1
Young leaf color	Green: 0; Yellow-green: 1; Brown-red: 2; Purple-red: 3; Purple: 4
Young leaf upper pubescence	Dense: 0; Sparse: 1; None: 2
Young leaf lower pubescence	Dense: 0; Sparse: 1; None: 2
Mature leaf color	Light green: 0; Medium green: 1; Dark green: 2; Purple: 3
Mature leaf upper pubescence	Dense: 0; Sparse: 1; None: 2
Mature leaf lower pubescence	Dense: 0; Sparse: 1; None: 2
Leaf shape	Broad ovate: 0; Ovate: 1; Narrow ovate: 2; Suborbicular: 3; Broad elliptic: 4; Elliptic: 5; Narrow elliptic: 6
Leaf apex shape	Caudate: 0; Acuminate: 1; Acute: 2; Abruptly acuminate: 3; Obtuse: 4
Leaf base shape	Cuneate: 0; Convex: 1; Rounded: 2; Cordate: 3
Leaf base angle	Acute angle: 0; Obtuse angle: 1; Reflex angle: 2
Leaf margin shape	Sharply serrate: 0; Bluntly serrate: 1; Doubly serrate: 2
Total leaf lobes	None: 0; Few: 1 (<40%); Moderate: 2 (40–70%); Many: 3 (70–100%); All: 4
Leaf lobe depth	None: 0; Shallowly lobed: 1; Moderately lobed: 2; Deeply lobed: 3
Anthocyanin pigmentation	None: 0; Weak: 1; Moderate: 2; Strong: 3
Petiole color	Green: 0; Red-green: 1; Red: 2; Purple: 3
Petiole pubescence	Dense: 0; Sparse: 1; None: 2
Stipule state	Deciduous: 0; Residual: 1; Persistent: 2
Midvein color	Yellow-green: 0; Red-green: 1; Purple-red: 2
Main venation	Pinnate straight veins: 0; Pinnate looped veins: 1; Pinnate ternate veins: 2
Lateral veins color	Yellow-green: 0; Red-green: 1; Purple-red: 2
Primary fabric	Monopodial: 0; Sympodial: 1; Monopodial proximally, sympodial distally: 2
Minor secondary	None: 0; Present: 1
Major secondary framework	Craspedodromous: 0; Semicraspedodromous: 1; Festooned semicraspedodromous: 2; Craspedodromous proximally, semicraspedodromous distally: 3
Connection pattern between major secondary and midvein	Decurrent: 0; Secondary veins at base decurrent: 1; Straight: 2; Curved: 3Decurrent: 0; Decurrent proximally: 1; Straight: 2; Curved: 3
Major secondary spacing	Regular: 0; Irregular: 1; Decreasing proximally: 2; Gradually increasing proximally: 3; Abruptly increasing proximally: 4
Variation of major secondary angle to midvein	Consistent: 0; Inconsistent: 1; Decreasing proximally: 2; Increasing proximally: 3
Intersecondary proximal course	None: 0; Parallel to major secondary veins: 1; Perpendicular to midvein: 2
Intersecondary distal course	None: 0; Reticulate or branched: 1; Parallel to major secondary veins: 2; Curved: 3
Intersecondary length	None: 0; Less than half the length of the proximal secondary veins: 1; More than half the length of the proximal secondary veins: 2
Intersecondary frequency	None: 0; Less than one per intercostal area: 1; Usually one per intercostal area: 2; More than one per intercostal area: 3
Intercostal tertiary fabric	Opposite percurrent: 0; Alternate percurrent: 1; Mixed percurrent: 2
Intercostal tertiary angle variability	Consistent: 0; Inconsistent: 1; Decreasing exmedially: 2; Increasing exmedially: 3
Epimedial tertiary fabric	Opposite percurrent: 0; Alternate percurrent: 1; Mixed percurrent: 2
Proximal course of the epimedial tertiary	Perpendicular: 0; Not perpendicular: 1
Quaternary fabric	Opposite percurrent: 0; Alternate percurrent: 1; Mixed percurrent: 2
Areole development	Poor: 0; Medium: 1; Good: 2; Excellent: 3
Freely ending veinlets branching	Mostly unbranched: 0; Mostly with one branch: 1; Dichotomous: 2; Dendritic: 3
Lateral vein angle	The angle between major secondary vein and midvein, unit: °
Major secondary quantity	Number of all massive secondary vein in the leaf
Leaf vein density	Total length of all veins per unit leaf area, unit: mm/mm^2^
Leaf length	The maximum distance from the leaf base to any point on the leaf margin, unit: cm
Leaf width	The maximum leaf width perpendicular to the leaf length, unit: cm
Leaf widest distance	The straight-line distance from the widest part of the leaf to the base, unit: cm
Petiole length	The straight-line distance from the base to the tip of the petiole, unit: cm
Leaf area	Surface area of a single leaf, unit: cm^2^
Leaf length/leaf width	The ratio of leaf length to width
Leaf widest distance/Leaf length	The ratio of leaf widest distance to leaf length
Leaf widest distance/Leaf width	The ratio of leaf widest distance to leaf width

## Data Availability

Data available on request from the authors.

## References

[1] Zhou T., Shen X.C., Zhou D.J., Fan J.J., Zhao M.M., Zhang W.X., Cao F.L. (2018). Advances in the Classification of Crabapple Cultivars. Acta Hortic. Sin..

[2] Wang J.R., Zhang W.X., Di C.Y., Lu X.J. (2022). Leaf Color and Pigments of 48 Ornamental Crabapple Germplasms Leaves. Fujian J. Agric. Sci..

[3] Qian G.Z., Tang G.G. (2005). A Review on the Plant Taxonomic Study on the Genus *Malus* Miller. J. Nanjing For. Univ. (Nat. Sci. Ed.)..

[4] Chu W.Y., Fan J.J., Zhang W.X. (2020). Phenological stability of ornamental crabapple and its response to temperature change. J. Nanjing For. Univ. (Nat. Sci. Ed.).

[5] Qian G.Z. (2005). Research on the Taxonomy of the Genus *Malus* Mill. Ph.D. Thesis.

[6] Li J.C., Liu J.Q., Gao X.F. (2022). A Revision of the Genus *Malus* Mill. (Rosaceae). Eur. J. Taxon..

[7] Liu B.B., Ren C., Kwak M., Hodel R.G.J., Xu C., He J., Zhou W.B., Huang C.H., Ma H., Qian G.Z. (2022). Phylogenomic Conflict Analyses in the Apple Genus *Malus* s.l. Reveal Widespread Hybridization and Allopolyploidy Driving Diversification, with Insights into the Complex Biogeographic History in the Northern Hemisphere. J. Integr. Plant Biol..

[8] Forte A.V., Ignatov A.N., Ponomarenko V.V., Dorokhov D.B., Savelyev N.I. (2002). Phylogeny of the Malus (Apple Tree) Species, Inferred from the Morphological Traits and Molecular DNA Analysis. Russ. J. Genet..

[9] Patzak J., Paprštein F., Henychová A., Sedlák J. (2012). Comparison of Genetic Diversity Structure Analyses of SSR Molecular Marker Data within Apple (*Malus* × *Domestica*) Genetic Resources. Genome.

[10] Kišek M., Jarni K., Brus R. (2021). Hybridisation of *Malus Sylvestris* (L.) Mill. with *Malus* × *Domestica* Borkh. and Implications for the Production of Forest Reproductive Material. Forests.

[11] Simo Santalla P., Chu N.T., Georges D. (2000). Characterisation of crabapple Clones by Isozyme Electrophoresis. Acta Hortic..

[12] Zhang W.X., Zhao M.M., Fan J.J., Zhou T., Chen Y.X., Cao F.L. (2017). Study on Relationship between Pollen Exine Ornamentation Pattern and Germplasm Evolution in Flowering Crabapple. Sci. Rep..

[13] Soltis E.D., Soltis P.S., Doyle J.J., Gaut B.S. (2000). Contributions of Plant Molecular Systematics to Studies of Molecular Evolution. Plant Molecular Evolution.

[14] Fan J.J., Wang Y., Hao Z.P., Peng Y., Ma J.Z., Zhang W.X., Zhao M.M., Zai X.M. (2024). Characteristics of Phenotypic Variation of Malus Pollen at Infrageneric Scale. Plants.

[15] Zhou T., Ning K., Zhang W.X., Chen H., Lu X.Q., Zhang D.L., El-Kassaby Y.A., Bian J. (2021). Phenotypic Variation of Floral Organs in Flowering Crabapples and Its Taxonomic Significance. Plant Biol..

[16] Wang B.Q., Wang Y.K., Shen K., Jiang Y., Wang L., Yang W.Q. (2022). Study on Venation Characteristics of 25 *Sorbus* Species. Wild Plant Resour..

[17] Blonder B., Violle C., Bentley L.P., Enquist B.J. (2011). Venation Networks and the Origin of the Leaf Economics Spectrum. Ecol. Lett..

[18] Tian J., Yu X.L., Li J.X. (2010). Characteristcs of the leave venation for species of *styrax* from Hunan and their significances on plant classification. J. Cent. South Univ. For. Technol..

[19] Shi X.G., Li Y.Q., Li C.R., Song X.H., Ye C. (2009). Leaf Architecture of *Eurya* and its Taxonomic Significance. Bull. Bot. Res..

[20] Cao L.M., Wang Z.X., Cao M., Liu J.H., Lin Q., Xia N.H. (2014). Leaf Venation and Its Systematic Significance in Sapindaceae of China. Plant Divers. Resour..

[21] Huang W.X. (2017). Leaf Architecture of Genus *Prunus* L. *sensulato* (*s. l.*) and Its Taxonomic Significance. Master’s Thesis.

[22] Tian C.F., Li M., Huang Y.J., Zhou Y., Wang X.R. (2022). Leaf venation characteristics of simple-leavedtaxa of *Sorbus* in China. Guihaia.

[23] Wang B.Q., Liu P.L., Shen K., Wang Y.K., Jiang Y., Wang L., Yang W.Q. (2021). Leaf Venation Patterns of Fourteen Species of *Berberis* in Shaanxi Province. Shaanxi For. Sci. Technol..

[24] Song J.Y., Luo T., Zhang N. (2019). Leaf Structure Characteristics of Five Species of *Cymbidium*. Plant Sci. J..

[25] Ji N.N. (2022). The Taxonomic Significance of Embryo Sac Development, Leaf Vein Type and Carpel Number to *Malus hupehensis* and Its Related Species. Master’s Thesis.

[26] Kumar C., Singh S.K., Pramanick K.K., Verma M.K., Srivastav M., Singh R., Bharadwaj C., Naga K.C. (2018). Morphological and Biochemical Diversity among the *Malus* Species Including Indigenous Himalayan Wild Apples. Sci. Hortic..

[27] Höfer M., Eldin Ali M.A.M.S., Sellmann J., Peil A. (2014). Phenotypic Evaluation and Characterization of a Collection of *Malus* Species. Genet. Resour. Crop Evol..

[28] Feng Y.Y., Li Q.Y., Huang J.H., Hu S.Q. (2021). Numerical classification of 25 color-leafed *Osmanthus fragrans* clones (cultivars). J. Nanjing For. Univ. (Nat. Sci. Ed.).

[29] Wang Y.N., Feng T.J., Sun L., Liu X.R., Liu Y.B., Wang P. (2025). Differences and influencing factors of understory vegetation species diversity between typical plantations and natural forests in the loess area of western Shanxi Province, northern China. J. Beijing For. Univ..

[30] Xu J.F., Zhang W.X., Zhu L.L., Jiang H., Sun T.T., Yu W.W. (2024). Phenotypic diversity analysis of fruit traits of 78 North American crabapple cultivars. J. Nanjing For. Univ. (Nat. Sci. Ed.).

[31] Koehne B.A.E. (1893). Deutsche Dendrologie.

[32] Rehder (1940). Manual of Cultivated Trees and Shrubs in North America.

[33] Yu D.J. (1984). Taxonomy of Deciduous Fruit Trees.

[34] Li Y.N. (2001). Research of Germplasm Resources of Malus Mill.

[35] Li Y.N. (1999). Progress in Research on the Origin and Evolution of Genus *Malus* in the World. J. Fruit Sci..

[36] Fiala J.L., Daniels G.S. (1994). Flowering Crabapples: The Genus Malus.

[37] Zheng Y., Qu X.L., Guo L., Sun F.Y., Mao Z.Q., Shen X. (2008). Advances on Ornamental Crabapple Resources. J. Shandong Agric. Univ. (Nat. Sci.).

[38] Guo L., Zhou S.L., Zhang Z.S., Shen X., Cao Y., Zhang D.L., Shu H.R. (2009). Relationships of Species, Hybrid Species and Cultivars in Genus *Malus* Revealed by AFLP Markers. Sci. Silvae Sin..

[39] Bodor-Pesti P., Taranyi D., Deák T., Nyitrainé Sárdy D.Á., Varga Z. (2023). A Review of Ampelometry: Morphometric Characterization of the Grape (*Vitis* Spp.) Leaf. Plants.

[40] Li D.S., Shi Z.M., Feng Q.H., Liu F. (2013). Response of leaf morphometric traits of *Quercus* species to climate in the temperate zone of the North-South Transect of Eastern China. Chin. J. Plant Ecol..

[41] Wang C., Li L., Ni X.L., Li J. (2018). Study on the Developmental Anatomy of Structures and AerenchymaFormation in *Potamogeton perfoliatus* Stems and Leaves. Acta Bot. Boreal.-Occident. Sin..

[42] Zhou L.Y., Wang Y.Q., Zhang L., Hu Z.M. (2009). Mathematic Classification of 46 Species in *Rhododendron* with the Morphologic Characters. Sci. Silvae Sin..

[43] Chu A.X., Yang Y.J., Tang G.G., Tong L.L. (2009). Studies on Numerical Taxonomy of the *Malus halliana* Koehne Cultivars in Henan. Acta Hortic. Sin..

[44] Roth Nebelsick A., Uhl D., Mosbrugger V., Kerp H. (2001). Evolution and Function of Leaf Venation Architecture: A Review. Ann. Bot..

[45] Peng G.Q., Xiong Y.J., Yin M.Q., Wang X.L., Zhou W., Cheng Z.F., Zhang Y.J., Yang D.M. (2022). Leaf Venation Architecture in Relation to Leaf Size Across Leaf Habits and Vein Types in Subtropical Woody Plants. Front. Plant Sci..

[46] Mollman R., Çiftçi A., Kaleli B.S., Erol O. (2023). Teasing out Elevational Trends in Infraspecific *Prunus* Taxa: A Vein Analysis Approach. Microsc. Res. Tech..

[47] Cho M.-S., Kim J.H., Yamada T., Maki M., Kim S.-C. (2021). Plastome Characterization and Comparative Analyses of Wild Crabapples (*Malus Baccata* and *M. Toringo*): Insights into Infraspecific Plastome Variation and Phylogenetic Relationships. Tree Genet. Genomes.

[48] Wang X.B., Cao Y., Guo W., Ma L., Liu X.L. (2023). Morphological Characteristics of Pollen from 44 Species of Subgen. Yulania. Acta Hortic. Sin..

[49] Zhou T., Zhang W.X., Zhang D.L., El-Kassaby Y.A., Fan J.J., Jiang H., Wang G.B., Cao F.L. (2020). A Binary-Based Matrix Model for *Malus* Corolla Symmetry and Its Variational Significance. Front. Plant Sci..

[50] Reich P.B., Wright I.J., Cavender-Bares J., Craine J.M., Oleksyn J., Westoby M., Walters M.B. (2003). The Evolution of Plant Functional Variation: Traits, Spectra, and Strategies. Int. J. Plant Sci..

[51] Pu J., Zhang J., Zhao C., Fan J.J., Jiang W.L., Zhang W.X., Wang G.P. (2019). Analysis and evaluation on flower color characteristics of the *Malus* ‘Purple Prince’ half- sib progenies. J. Nanjing For. Univ. (Nat. Sci. Ed.).

[52] Fan J.J., Zhang W.X., Zhou T., Zhang D.D., Zhang D.L., Zhang L., Wang G.B., Cao F.L. (2018). Discrimination of *Malus* Taxa with Different Scent Intensities Using Electronic Nose and Gas Chromatography–Mass Spectrometry. Sensors.

[53] Zhang L.L., Mao Y.F., Zhang C.H., Zhang D.J., Shen X. (2019). A New Ornamental Crabapple Cultivar ‘Duojiao’. Acta Hortic..

[54] Ge H.J., Huang Y., Wan S.W., Zhang R.F., Ma R.Q., Sun J.L., Sha G.L. (2022). *Malus* ‘Datang Tingliang’: A new ornamental crabapple cultivar. J. Nanjing For. Univ. (Nat. Sci. Ed.).

[55] Li N., Zhang W.X., Jiang H., Zhang Q.Q., Zhao P.P. (2021). Changes of Leaf Color and Dynamics of Pigment Componentsin Ornamental Crabapple. North. Hortic..

[56] Han W.X., Jiang H., Bian J., Yun J.H., Sun Y.Y., Zhang W.X., Peng Y. (2020). Leaf color change and its correlation with pigment content in 10 ornamental crabapple varieties in spring. J. Zhejiang Univ. Agric. Life Sci..

[57] Ellis B., Daly D., Hickey L., Johnson K., Mitchell J., Wilf P., Wing S. (2009). Manual of Leaf Architecture.

[58] IBM Corp (2019). IBM SPSS Statistics forWindows.

[59] R Core Team (2023). R: A Language and Environment for Statistical Computing.

[60] Wickham H. (2016). Ggplot2: Elegant Graphics for Data Analysis.

[61] Wei T., Simko V. (2021). R Package “Corrplot”: Visualization of a Correlation Matrix. https://github.com/taiyun/corrplot.

[62] Xiao N. (2024). Ggsci: Scientific Journal and Sci-Fi Themed Color Palettes for “Ggplot2”. https://CRAN.R-project.org/package=ggsci.

